# Supplementary material for: Direct mapping of kidney function by DCE-MRI urography using a tetrazinanone organic radical contrast agent
Source: Nat Commun. 2023 Jul 5;14:3965. doi: 10.1038/s41467-023-39720-x (PMC10322853; doi:10.1038/s41467-023-39720-x)
Supplement: Supplementary file 1 — Supplementary Information [file 41467_2023_39720_MOESM1_ESM.pdf]

Supplementary Information

Direct Mapping of Kidney Function By MRI-Based Urography Using a Tetrazinanone Organic Radical Contrast Agent

Nicholas D. Calvert[1], Alexia Kirby[2], Mojmír Suchý[1], Peter Pallister[3], Aidan A. Torrens[1], Dylan Burger[4], Gerd Melkus[5,6], Nicola Schieda[6], Adam J. Shuhendler[1,2,7]

[1]Department of Chemistry and Biomolecular Sciences, University of Ottawa, 150 Louis Pasteur Pvt., Ottawa, Ontario, Canada, K1N 6N5
[2] Department of Biology, University of Ottawa, 150 Louis Pasteur Pvt., Ottawa, Ontario, Canada, K1N 6N5
[3]Department of Chemistry, Carleton University, 1125 Colonel By Dr., Ottawa, Ontario, Canada, K1S 5B6
[4] Kidney Research Center, Ottawa Hospital Research Institute, University of Ottawa, 501 Smyth Rd, Ottawa, Ontario, Canada K1H 8L6
[5]Dept. Medical Imaging, The Ottawa Hospital, 501 Smyth Rd, Ottawa, Ontario, Canada K1H 8L6.
[6]Dept. Radiology, University of Ottawa, 501 Smyth Rd, Ottawa, Ontario, Canada K1H 8L6.
[7]University of Ottawa Heart Institute, 40 Ruskin St., Ottawa, Ontario, Canada, K1Y 4W7

1. General Reagents:

All cell culture reagents and consumables were purchased from ThermoFisher, with the exception of the Epithelial Cell Media and Epithelial Cell Growth Supplement, which were purchased from ScienCell.

2. Experimental Procedures:

All EPR spectra were acquired on a Bruker EMX plus EPR at room temperature. All MRI acquisitions were performed on a 3 T pre-clinical MRI scanner (MR Solutions, Ltd.). For all MRI image data analysis, only relevant slices of the tissue of interest were included in analysis (i.e., scans for kidney region only used slices with the kidney visible). All data processing, mapping, and quantity generation was done using a custom program written in MATLAB 2020A®. A copy of this program is available from the authors upon request. GraphPad Prism 9.5 was used to generate all graphs, graphical figures, and statistical results.

2.1 Phantom MRI, determination of glucoverdazyl longitudinal relaxivity and evaluation of temperature-dependence of relaxation rates.

Contrast agent samples were prepared in 1× PBS in standard NMR tubes, which were then inserted into a 50 mL Falcon tube containing ultrasound gel, comprising the MRI phantom. The MRI phantom was placed into a 38-mm-diameter send and receive volume coil and inserted into the MRI scanner. A multislice Rapid Imaging with Refocused Echoes (RARE) pulse sequence was implemented for evaluation of phantoms using the following parameters for T_1-weighted imaging: slice thickness of 5 mm, FOV of 40 × 40 mm, averages = 3, matrix size = 96 × 96, TE_{eff} = 11 ms, echo spacing = 7 ms, TR = 720 ms, and acquisition time of 2 minutes 16 seconds. For T_2-weighted imaging, all parameters were the same as for T_1-weighted images, except TE_{eff} = 68 ms and TR = 4800 ms, and acquisition time was 7 minutes 28 seconds.

For relaxivity measurements, the same imaging phantom was used with contrast agent concentrations of 1 mM or 0.1 mM contrast agent in 0% to 22.5% HSA in PBS, or 3 mM contrast

agent in PBS of pH 3, 7, or 11. Contrast agent concentration following imaging was verified by electron paramagnetic spectroscopy. To measure the longitudinal relaxation rate (R_1), an inversion recovery RARE sequence was implemented with the following parameters: Slice thickness of 5 mm, FOV of 50 $\times$ 50 mm, average = 1, matrix size = 96 $\times$ 96, TE_{eff} = 17 ms, TR = 5,000 ms, TI = 50, 75,100, 150, 200, 250, 300, 400, 600, 800, 1200, 2400, and 4800 ms, and acquisition time of 2 minutes 30 seconds *per* TI. Longitudinal relaxation rates were extracted using the mapping MATLAB 2 routine written by J. Barral, M. Etezadi-Amoli, E. Gudmundson, and N. Stikov (2009), and modified by J. Rioux (2022). The longitudinal relaxivity (r_1) was extracted from the slope of the plot of $1/T_1$ vs. contrast agent concentration.

High field NMR relaxation measurements were performed at 7.05 T ($v_0(^1H)$ = 300.15 MHz) on a Bruker Avance spectrometer. Samples were dissolved in PBS buffer in a mixture of 80:20 H_2O:D_2O. D_2O was used as a field lock. ^1H NMR experiments on pure water at high field with a high Q probe will produce significant radiation damping. So, commonly used inversion recovery experiments for determining T_1 will result in artificially smaller T_1 measurements due to small amounts of transverse magnetization produced with slightly imperfect inversion pulses. This was the case for these samples (data not shown). To alleviate this outcome, a saturation recovery experiment was used to determine T_1. A 3 s CW saturation pulse was used on-resonance prior to the variable delay and read pulse to collect time-arrayed saturation recovery data. Ten – twelve time points were collected at each temperature. T_2 data was collected using a Car-Purcell-Meiboom-Gill (CPMG) sequence with a 20 ms total echo time and an appropriate number of loops to ensure complete loss of signal at each temperature. Eleven time points were collected at each temperature. A relaxation delay of at least 5*T_1 was used for T_1 and T_2 measurements at each temperature. T_1 data was fit using Bruker Topspin 4.1.4 and T2 data was fit using GNAT. [1]

2.2 Glucoverdazyl stability measurements.

The EPR was tuned to a 5 mM sample of glucoverdazyl in PBS prior to any of the stability measurements. Once tuned, glucoverdazyl test solutions were prepared (5 mM in either 10 mM glutathione (GSH), 10 mM hydrogen peroxide (H_2O_2), or 3 mM in PBS of pH = 3, 7, or 11). A single spectrum was acquired and the peak height of the most intense peak for either compound was locked. EPR scans were then acquired every 5 s for 1.5 hr (H_2O_2, glutathione, and pH) to measure percent change in the activity.

Glucoverdazyl interaction with hydroxyl radicals generated through Fenton reactions was evaluated through $FeCl_2$ to $FeCl_3$ oxidation catalyzed by H_2O_2. A 20 mM ascorbate buffer (pH = 4.5) containing 1 mM $FeCl_2$ and 10 mM 5,5-dimethyl-1-pyrroline-N-oxide (DMPO). This solution was sparged with N_2 for 30 min and aliquoted into vials containing glucoverdazyl or TEMPO to make a 5 mM concentration of either radical. Immediately prior to EPR scan, 10 mM H_2O_2 was added to the solution. After mixing, the EPR activity was measured, with measurement repeated after 1 hr. Aliquots of the 1 hr solution were taken and absorbance was measured at 330 nm to determine the amount of $FeCl_3$ produced by $FeCl_2$ oxidation by H_2O_2. Absorbance of TEMPO and glucoverdazyl without Fe(II) were subtracted from the oxidized solutions of each to account for inherent absorbance of these molecules.

Glucoverdazyl interaction with superoxide was evaluated by xanthine (X)/xanthine oxidase (XO) reaction. Solutions of either TEMPO or glucoverdazyl (1.25 mM) in PBS were made containing either 10 mM X, 0.4 U XO, or both. These solutions were incubated under cell culture conditions for 30 min before EPR activity of the solution was measured. Aliquots of the solution were taken and absorbance was measured at 293 nm to determine the production of

uric acid, the product of the X/XO reaction. Absorbance of TEMPO and glucoverdazyl without X, XO, or X/XO were subtracted from the oxidized solutions of each to account for inherent absorbance of these molecules.

2.3 Assessment of human serum albumin binding.

Human serum albumin binding assays for both binding and site specificity were adapted from methods by Caravan *et al.,* 2002. [2] A solution of 4.5% w/v human serum albumin (HSA) containing 3 mM glucoverdazyl was prepared. The solution was serially diluted with a 4.5% HSA solution in PBS. The dilutions were incubated under cell culture conditions for 30 min. Afterwards, free glucoverdazyl was separated from bound glucoverdazyl through ultracentrifugation separation using Centrifree® Ultrafiltration filters (2000 xg, 20 min). The unbound glucoverdazyl in the filtrate was quantified by HPLC using the protocol described in Supplementary section 3.2, after developing a glucoverdazyl HPLC standard curve in PBS. The concentration of bound glucoverdazyl was calculated by subtracting the added concentration of glucoverdazyl from the HPLC determined concentration of free glucoverdazyl.

Site specificity of glucoverdazyl binding was determined by displacement of site-specific fluorophores (dansylamide, site I; dansylglycine, site II). A solution of 4.5% w/v HSA with 3 mM glucoverdazyl was prepared containing 50 mM dansylamide or dansylglycine. These solutions were serially diluted with a 4.5% w/v HSA solution containing 50 mM of either probe but no glucoverdazyl. Another set of solution containing the glucoverdazyl dilutions in HSA but with neither probe was also prepared. These dilutions were incubated under cell culture conditions for 30 min. The dilutions were aliquoted in a black-walled 96-well plate. Fluorescence was measured (ex. 365 nm/em. 480 nm) for each well. Intensity values for glucoverdazyl without probe were subtracted from the same concentration containing probe to account for absorbance incurred by the glucoverdazyl molecule. Corrected fluorescence values were compared to the either fluorescent probe in HSA containing no glucoverdazyl to determine the degree of probe displacement from HSA caused by glucoverdazyl binding.

2.4 Assessment of cell viability in H460 cells.

Large-cell lung cancer cells (H460) were grown in RPMI-1640 (RPMI) media supplemented with 10% fetal bovine serum (FBS) and 1% penicillin-streptomycin (P/S) until 80% confluent, at which point they were passaged. Cells were passaged three times before being seeded in to a 6-well plate and grown until 80% confluent. Cells were seeded to have triplicate wells of each condition. Cells were then incubated in their regular media supplemented with 0 mM, 2.5 mM, 5 mM, or 10 mM glucoverdazyl, for either 4 hrs or 24 hrs. At the respective time points, the media was aspirated and cells were washed three times with 37°C Dulbecco's phosphate-buffered saline (PBS). Afterwards, cells were lifted with trypsin-EDTA, centrifuged at 400 xg (5 min, 4°C), aspirated, then resuspended in a 1 mL PBS solution containing 0.2 µM calcein-acetoxymethyl ester (fluorescently staining live cells green) and 16 µM ethidium homodimer-1 (fluorescently staining dead cells red).

Live and dead cell populations were counted by flow cytometry (Beckman-Coulter Gallios Flow Cytometer) using a 488 nm excitation with a 525 nm / 40 nm bandpass filter for calcein-acetoxymethyl ester (live cells, green) and a 620 nm / 20 nm bandpass filter for ethidium homodimer-1 (dead cells, red). After performing, the viable cell population for each condition was determined by comparing the total number of singly-stained, calcein-AM-positive cell

counts to the combined total of cells that were singly-stained as positive for live or dead using Kaluza analysis software (Beckman-Coulter).

2.5 Assessment of viability of human renal proximal tubule (hRPT) cells.

hRPT cells were grown in Epithelial Cell Media (EpiMEM) media supplemented with 10% FBS, 1% P/S, and epithelial cell growth supplement (EpiCGS) until 80% confluent, at which point they were passaged. Cells were passaged three times before being seeded in to a 6-well plate and grown until 80% confluent. Cells were seeded to have triplicate wells of each condition. Cells were then incubated in their regular media supplemented with regular media, 10 mM glucoverdazyl, 10 mM 5,5-dimethyl-1-pyrroline *N*-oxide (DMPO, a nitrone spin trap), or 10 mM (2,2,6,6-Tetramethylpiperidin-1-yl)oxyl (TEMPO, a nitroxy radical), for either 4 hrs or 24 hrs. At the respective time points, the media was aspirated and cells were washed three times with 37°C Dulbecco's phosphate-buffered saline (PBS). Afterwards, cells were lifted with trypsin-EDTA, centrifuged at 400 xg (5 min, 4°C), aspirated, then resuspended in a 1 mL PBS solution containing 0.2 µM calcein-acetoxymethyl ester (fluorescently staining live cells green) and 16 µM ethidium homodimer-1 (fluorescently staining dead cells red).

Live and dead cell populations were counted through flow cytometry (Beckman-Coulter Gallios Flow Cytometer) using a 488 nm excitation with a 525 nm / 40 nm bandpass filter for calcein-acetoxymethyl ester (live cells, green) and a 620 nm / 20 nm bandpass filter for ethidium homodimer-1 (dead cells, red). After performing, the viable cell population for each condition was determined by comparing the total number of singly-stained, calcein-AM-positive cell counts to the combined total of cells that were singly-stained as positive for live or dead using Kaluza analysis software (Beckman-Coulter).

2.6 Evaluation of glucoverdazyl uptake in hRPT and hepatocarcinoma (HEPG2) cells.

hRPT cells were grown in Epithelial Cell Media (EpiMEM) media supplemented with 10% FBS, 1% P/S, and epithelial cell growth supplement (EpiCGS) until 80% confluent, at which point they were passaged. Cells were passaged three times before being seeded in to 6-well plates and grown until 80% confluent. Cells were seeded to have triplicate wells of each condition. Cells were then incubated in their regular media supplemented with regular media or 10 mM glucoverdazyl, and incubated for 24 hrs. The media was aspirated and cells were washed three times with 37°C Dulbecco's phosphate-buffered saline (PBS). Afterwards, cells were lifted with trypsin-EDTA, centrifuged at 400 xg (5 min, 4°C), aspirated, then resuspended in 100 µl of PBS. The concentrated cell solutions were transferred to EPR tubes. A 1 µl aliquot was retained and diluted to obtain the number of cells in each solution.

HEPG2 cells were grown in RPMI supplemented with 10% FBS and 1% P/S until 80% confluent, at which point they were passaged. Cells were passaged three times before being seeded in to 6-well plates and grown until 80% confluent. Cell media was changed to DMEM supplemented with 10% FBS and 1% P/S, containing no glucose for 1 hr. The media was replaced after 1 hr containing 0 or 10 mM glucoverdazyl. The cells were then incubated for 24 hrs. The media was aspirated and cells were washed three times with 37°C with PBS. Cells were then lifted with trypsin-EDTA, centrifuged at 400 xg (5 min, 4°C), aspirated, then resuspended in 100 µl of PBS. The concentrated cell solutions were transferred to EPR tubes. A 1 µl aliquot was retained and diluted to obtain the number of cells in each solution.

The EPR was tuned to a 5 mM solution of freshly prepared glucoverdazyl in PBS, then samples were measured by the EPR. Concentration was measured against a previously determined standard curve, then normalized to the number of cells previously determined to obtain nM glucoverdazyl per cell.

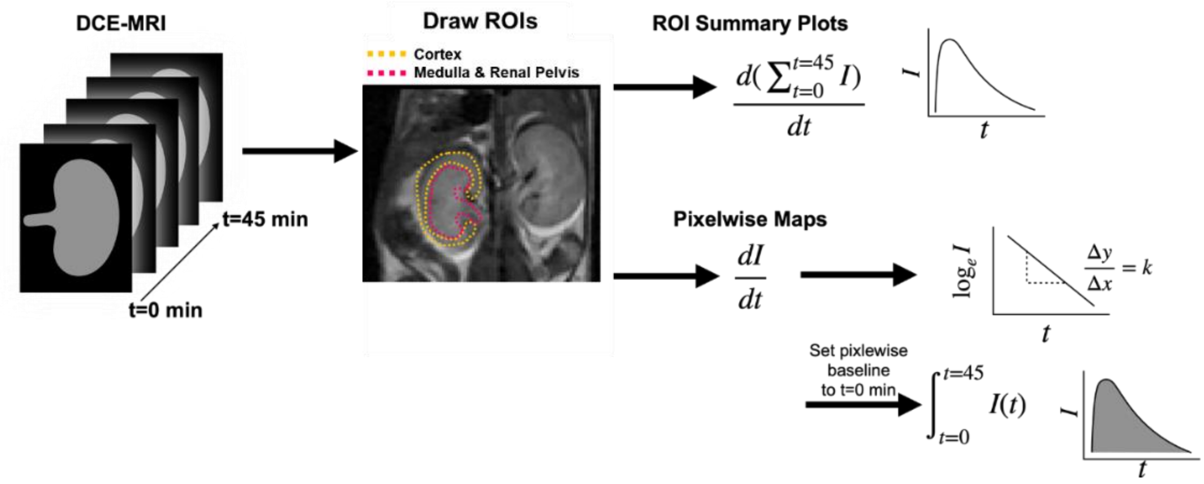

S1. Illustration of DCE-MRI data acquisition and image mapping workflow.

2.7 Statistics

Statistical analyses for experiments are reported in the corresponding methods section. Normality was assumed where appropriate for all data sets. Prior to ANOVA, Levene's test was use to confirm equal variance, and visual quantile-quantile plot analysis was used to confirm homoscedasticity.

3. Characterization Data

3.1 NMR Spectra

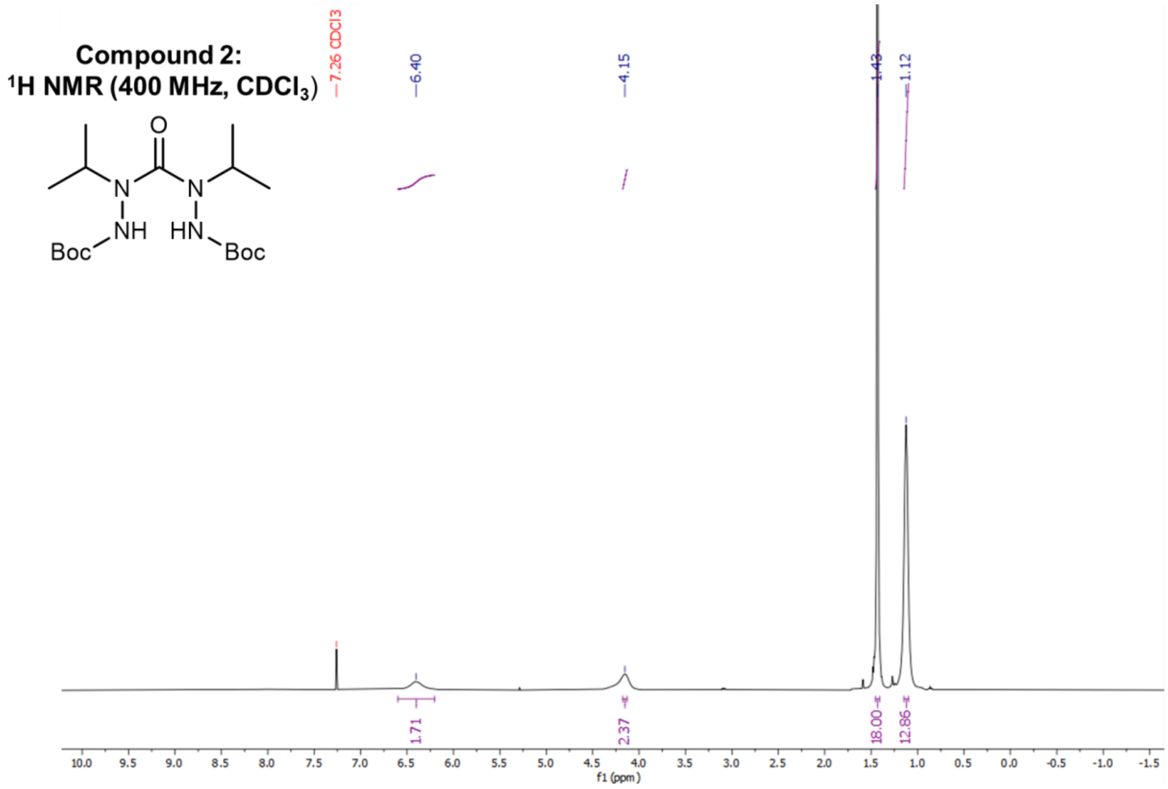

S2. ^{11}H NMR spectrum of compound 2 in $CDCl_3$.

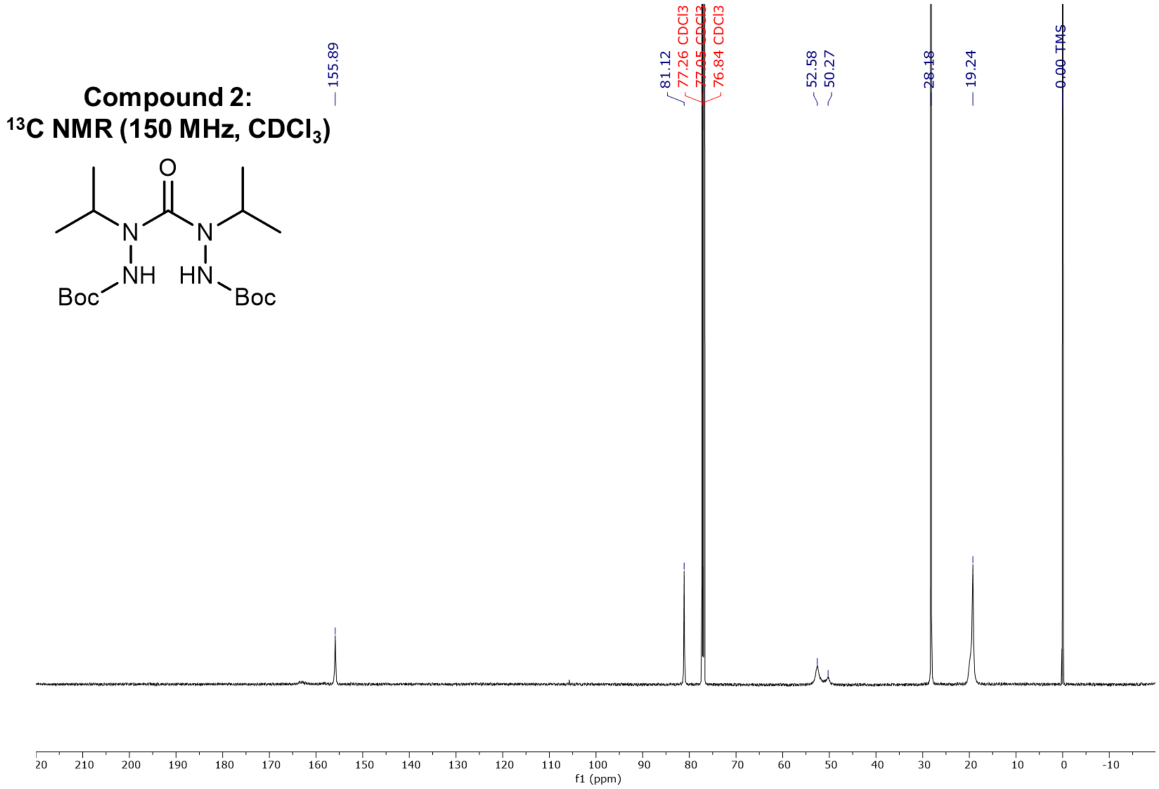

S3. ^{13}C NMR spectrum of compound 2 in $CDCl_3$.

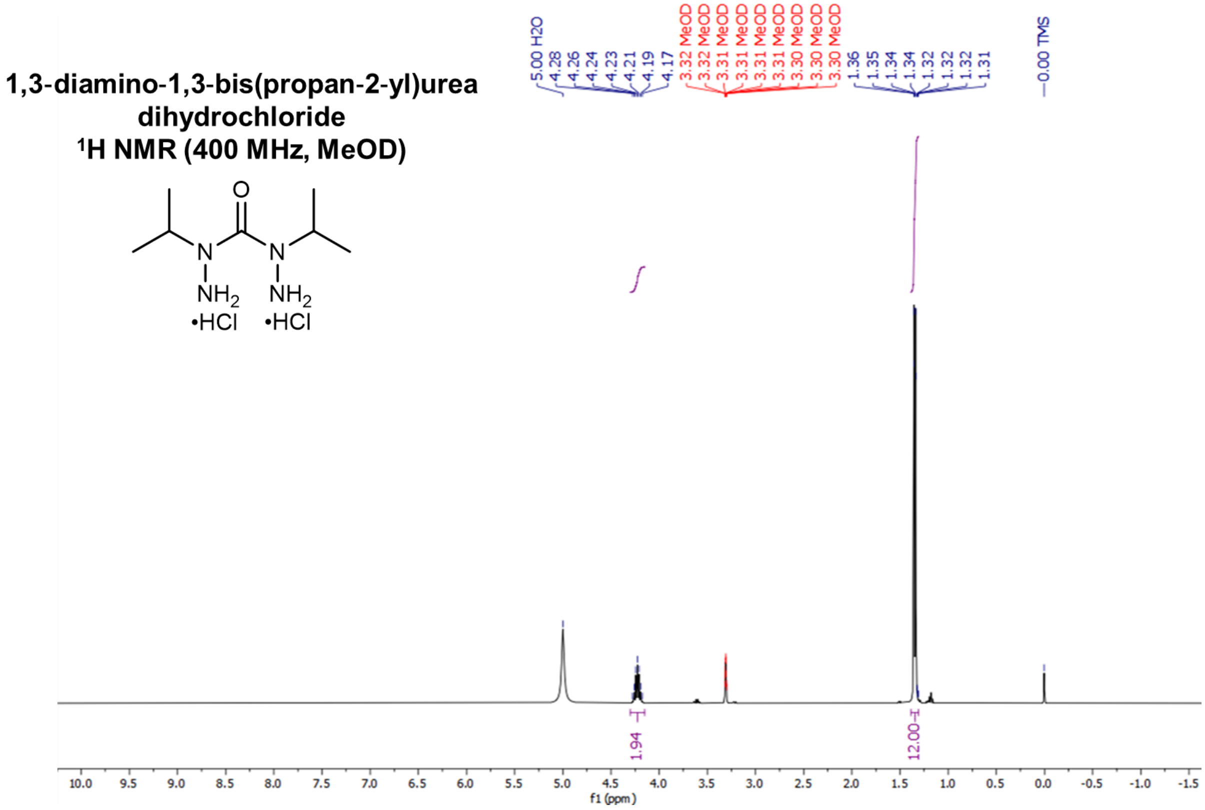

S4. ^{1}H NMR spectrum of 1,3-diamino-1,3-bis(propan-2-yl)urea dihydrochloride in MeOH-D_4.

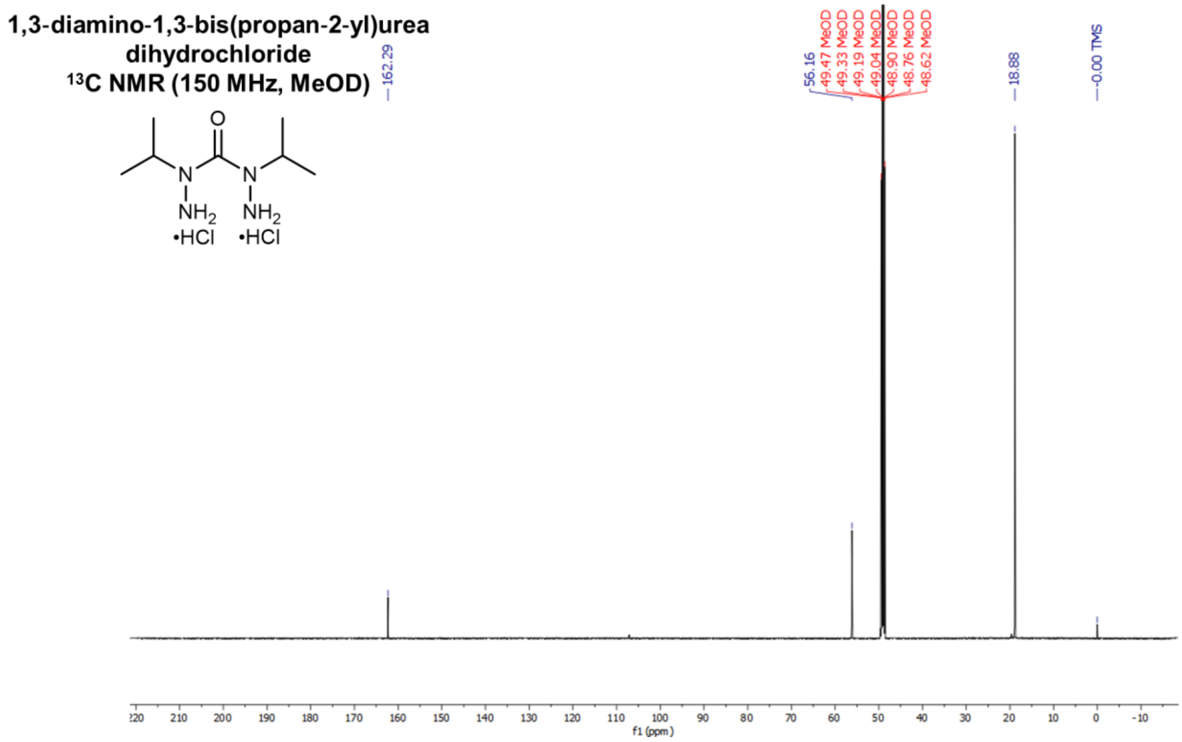

S5. ^{13}C NMR spectrum of 1,3-diamino-1,3-bis(propan-2-yl)urea dihydrochloride in MeOH-D_4.

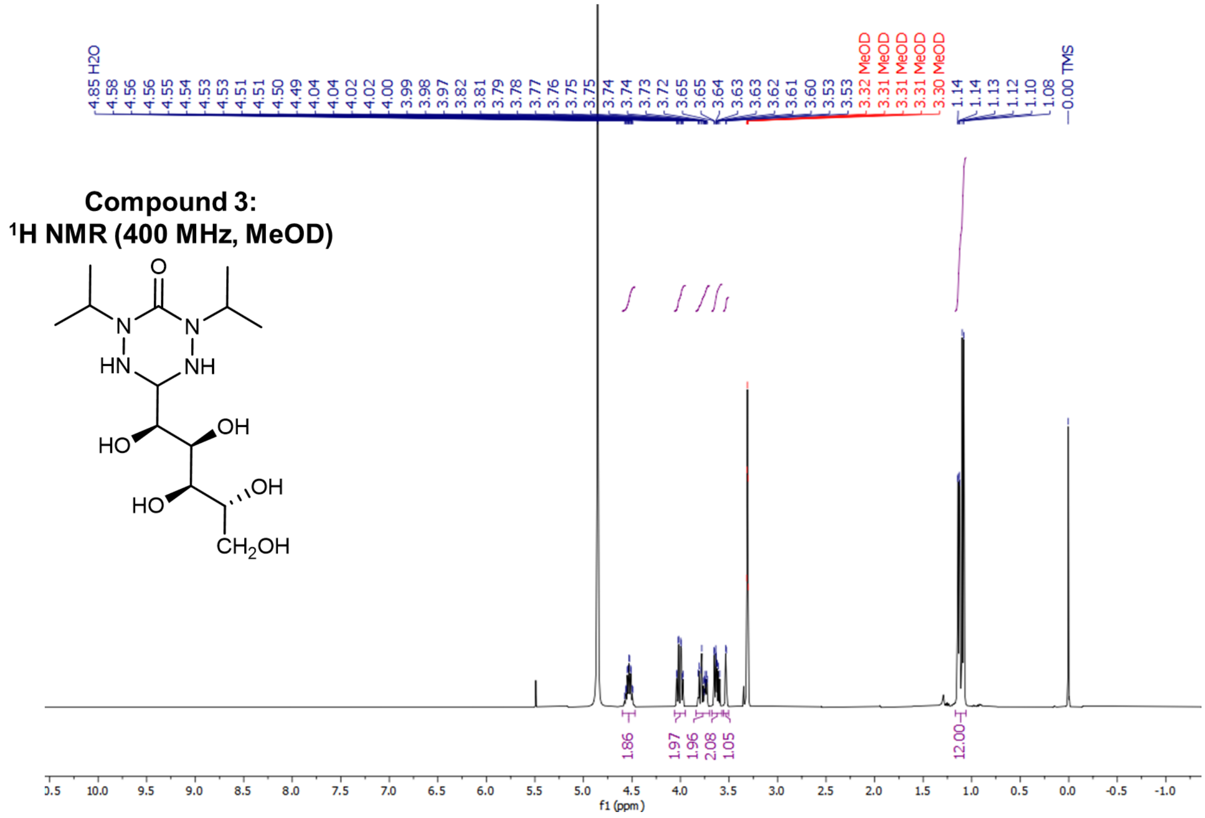

S6. ^{1}H NMR spectrum of compound 3 in MeOH-D_4.

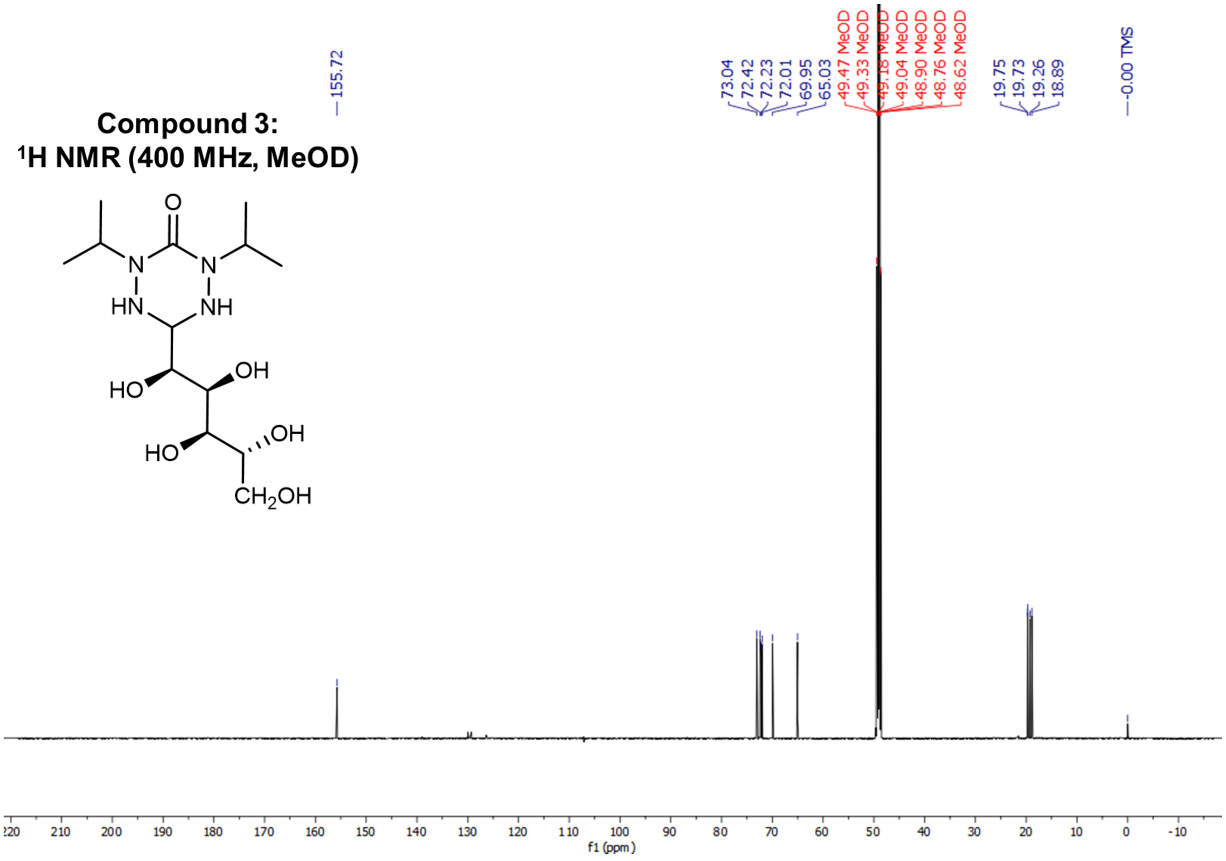

S7. ^{13}C NMR spectrum of compound 3 in MeOH-D$_4$.

3.2 High-performance liquid chromatography traces of 3 and 4.

In order to verify the completion of radicalization from compound **3** to **4**, we used HPLC traces in conjunction with HRMS and EPR. HPLC analysis was performed on a Prominence HPLC system with diode array detector (Shimadzu North America) using a Luna® C_{18} 100 Å column (4.6 mm x 250 mm, 5µm particle size). A 20 min HPLC gradient with mobile phase consisting of a 0.5% trifluoroacetic acid (TFA) in H_2O gradually increasing from 1% to 100% with AcN containing 0.5% TFA min was used at a flow rate of 1 mL/min. Retention time (t_R) of compound **3** was consistently 10.5 min, while after radicalization and loss of two protons from the verdazyl ring, the t_R increased to 11.8 min. As well, radicalization induced greater absorbance at 452 nm for compound **4**, while no signal at this wavelength is observed in the non-radical compound **3**.

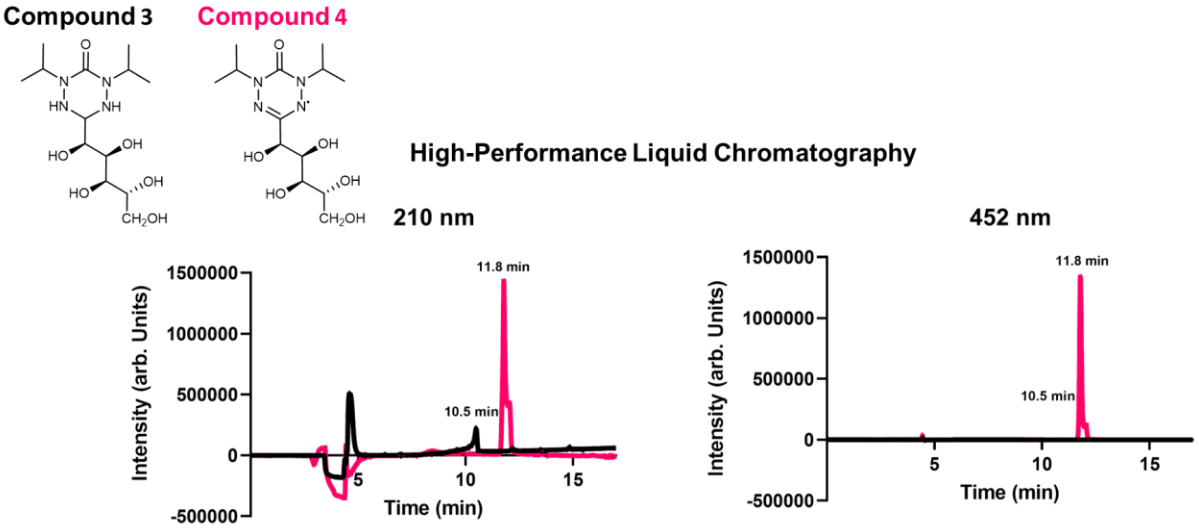

S8. High performance liquid chromatography traces of compounds 3 and 4 to verify radical activity of the compound after radicalization step.

3.4 Temperature-dependence Of Relaxation Rate

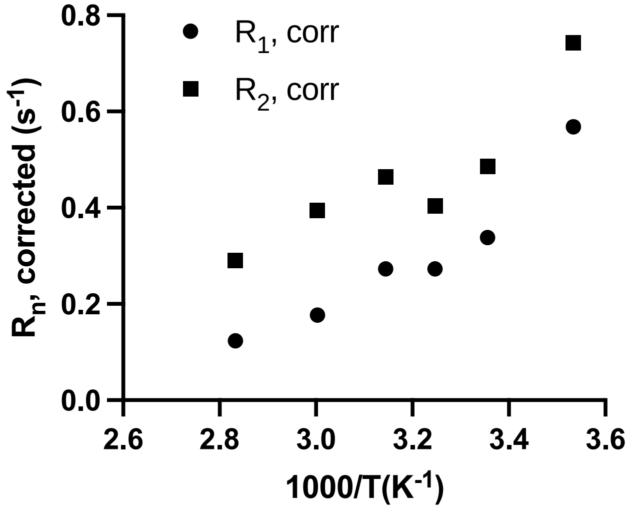

S9. **Temperature-dependency of glucoverdazyl relaxivity.** Temperature-dependence of the longitudinal (circle) and transverse (square) relaxation rates of glucoverdazyl, corrected for buffer effects, at 7.0 T.

3.5 Stability Studies

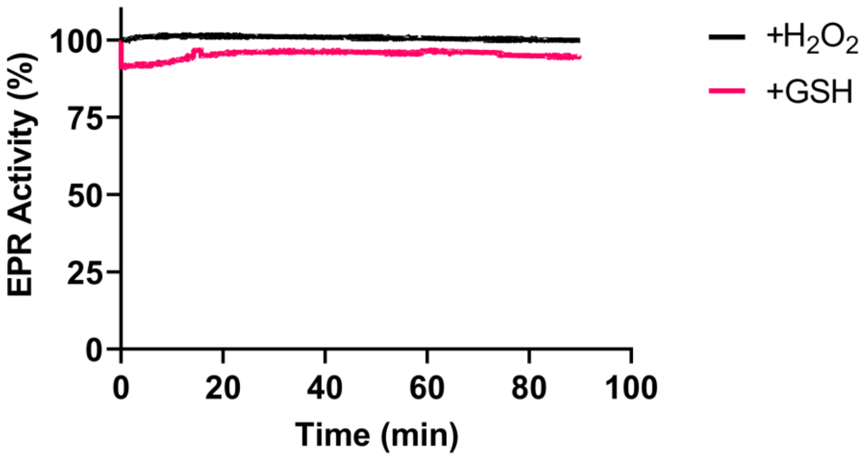

S10. Glucoverdazyl stability in the presence of known oxidizers and reducers. Twenty millimolar of either hydrogen peroxide (H_2O_2) or reduced glutathione (GSH) were added to a solution of 5 mM glucoverdazyl in phosphate-buffered saline (PBS). These solutions were monitored by an EPR that was previously tuned to a 5 mM sample of glucoverdazyl in PBS. Solutions were monitored for 90 min to determine if any loss of radical activity occurred by either oxidation or reduction of glucoverdazyl.

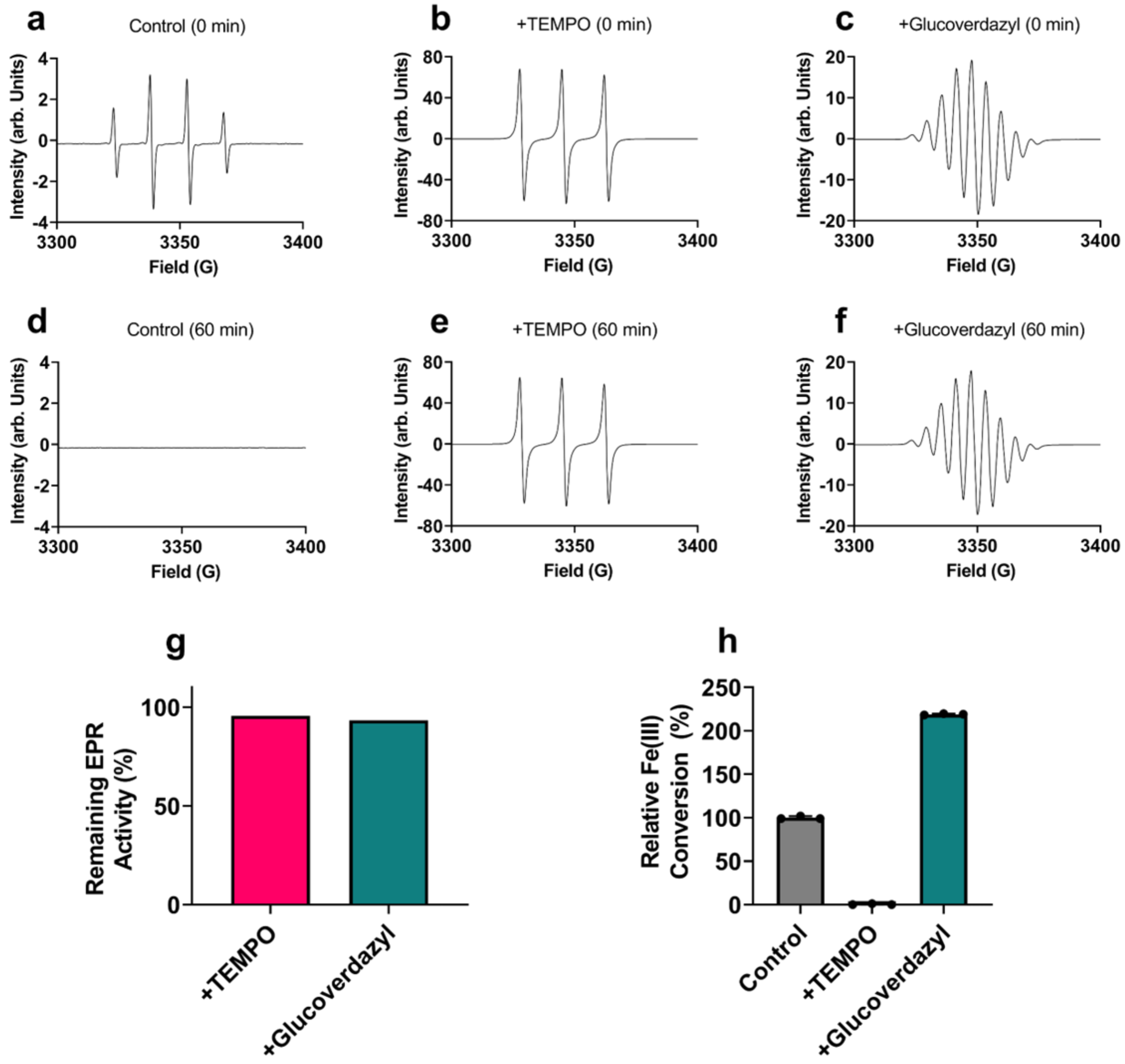

S11. Interactions of TEMPO and glucoverdazyl with Fenton chemistry by H_2O_2 mediated oxidation of $FeCl_2$ to $FeCl_3$. EPR spectra of 10 mM DMPO alone or with 5 mM of either TEMPO or glucoverdazyl before (A, B, C) and 1 hr after (D, E, F) the addition of 10 mM H_2O_2 to initiate oxidation of $FeCl_2$ to $FeCl_3$. G) The change in maximum EPR signal before and 1 hr after oxidation reaction. H) Conversion of Fe(II) to Fe(III) determined by change in absorbance at 330. Data are presented as means ± standard deviation of n = 3 technical replicates.

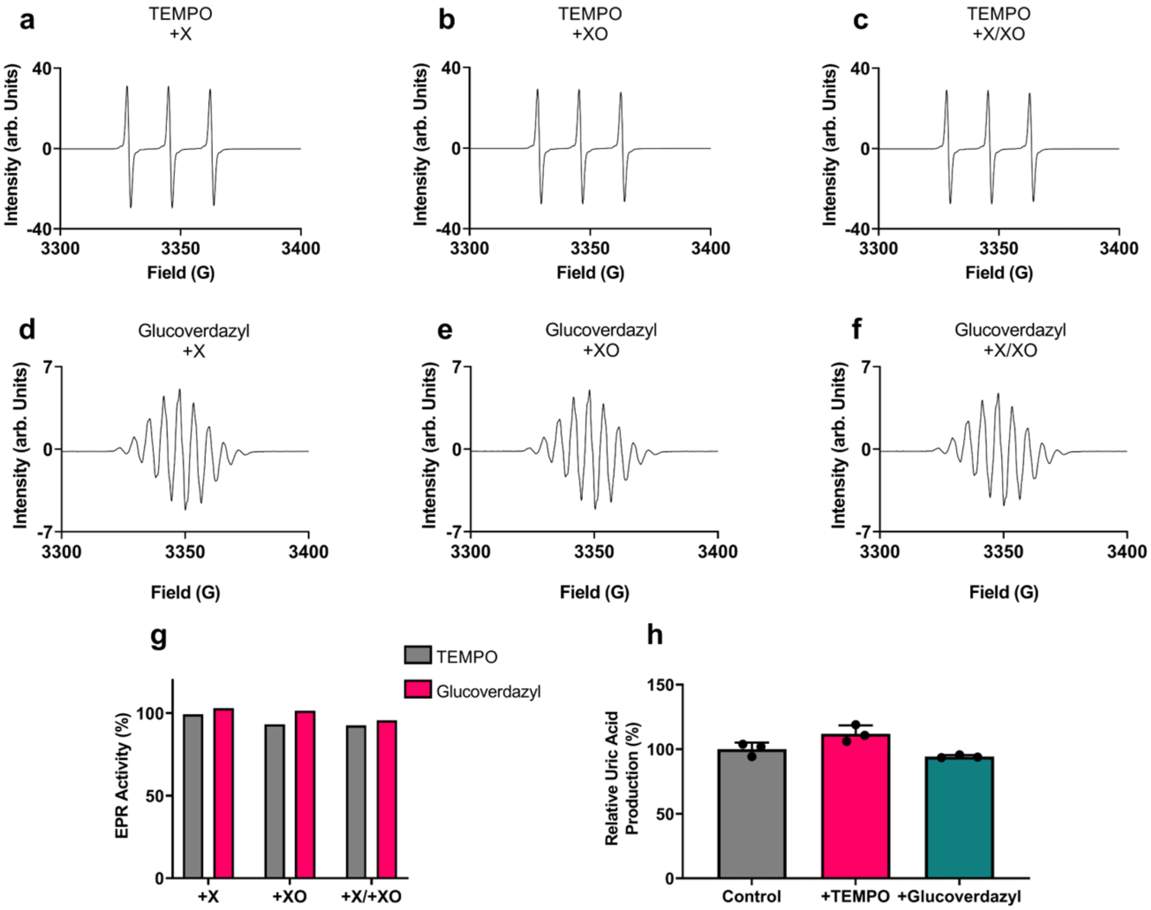

S12. Interactions of TEMPO and glucoverdazyl with superoxide produced by xanthine oxidase. EPR spectra of 1.25 mM TEMPO containing 10 mM xanthine (X, S12. A), 0.4 U xanthine oxidase (XO, S12. B), or both (X/XO, S12. C). S12. D, E, and F are the same but with 1.25 mM glucoverdazyl. The change in EPR maxima compared to the tuned radical is shown in S12. G Relative uric acid production as a result of the X/XO reaction is shown in S12. H by change in absorbance at 293 nm. Data are presented as means ± standard deviation of n = 3 technical replicates.

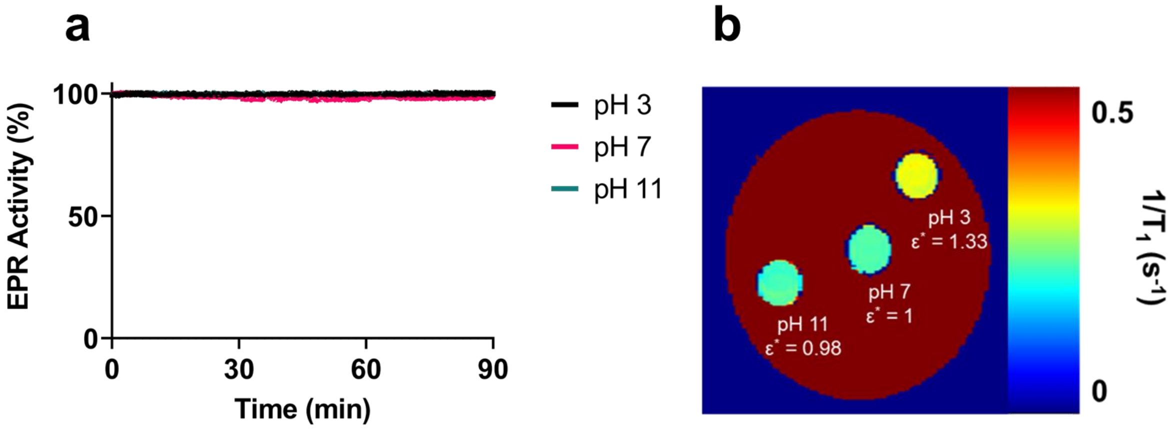

S13. Change in glucoverdazyl radical stability and relaxation by pH. A) Change in EPR activity of a 3 mM glucoverdazyl solution in PBS at pH 3, 7, or 11. B) Relaxation of a 3 mM glucoverdazyl solution in PBS at pH 3, 7, or 11. The change in relaxation compared to pH = 7 is shown as ε^* ($\frac{1}{T_{1HSA}} / \frac{1}{T_{1PBS}}$).

3.6 Human Serum Albumin Binding.

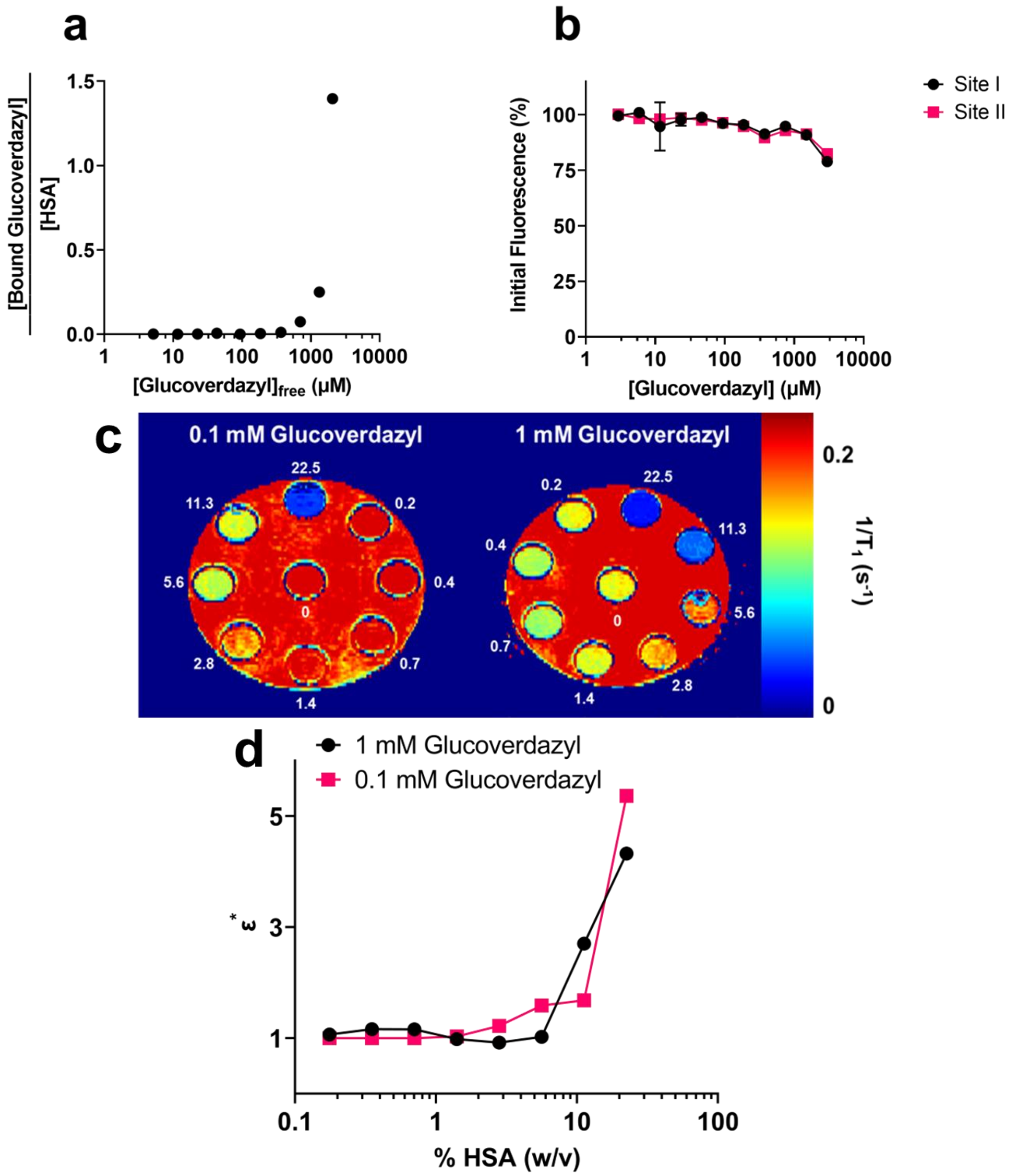

S14. Glucoverdazyl binding to human serum albumin (HSA). A) Glucoverdazyl fraction bound to a 4.5% w/v solution of HSA in PBS as determined by HPLC following ultrafiltration of a glucoverdazyl/HSA solution, separating bound from free fractions. B) Occupation of HSA binding site I or II by glucoverdazyl as determined fluorescently by displacement of site I or site II specific dansylamide or dansylglycine, respectively. Data are presented as means ± standard deviation of n = 3 technical replicates. C) Relaxivity maps of NMR tubes filled with glucoverdazyl (0.1 mM or 1 mM) in either PBS or varying concentrations of HSA (numerically listed in the image as w/v) as determined by MRI at 3 T. D) graphical representation of relaxation enhancement, ε^* ($\frac{1}{T_{1HSA}}/\frac{1}{T_{1PBS}}$) in the presence of HSA.

3.7 Cytotoxicity Evaluations of Glucoverdazyl.

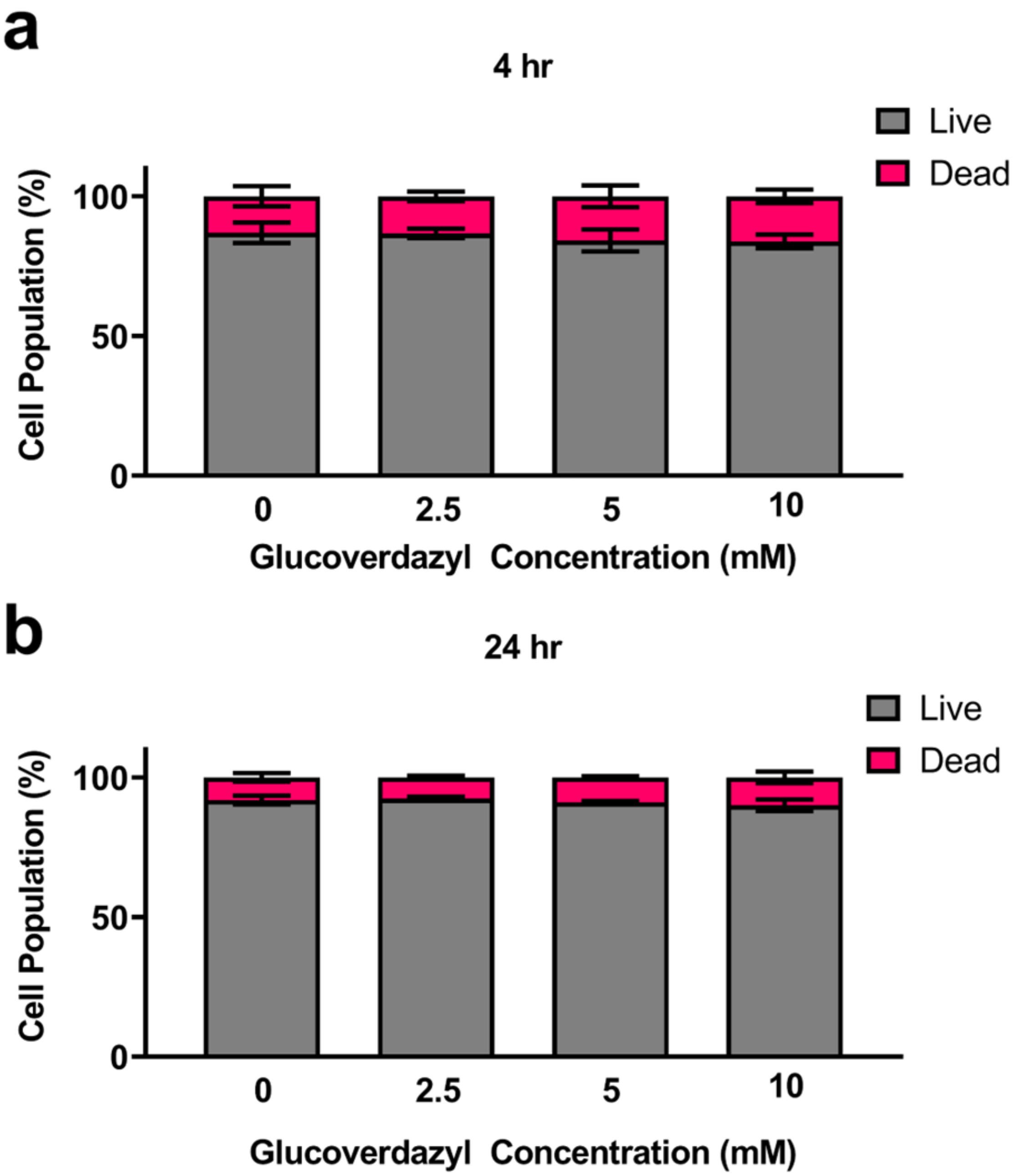

S15. Cytotoxicity evaluation of glucoverdazyl at different concentrations in H460 cells. Cellular viability after 4 hr (A) and 24 hr (B) incubations with the respective concentration. Data are represented as means ± SD of n = 3 replicates. Statistical analysis was done by one-way ANOVA followed by a Tukey post-hoc test. An example gating strategy used to obtain this data can be seen in Supplementary Figure 23.

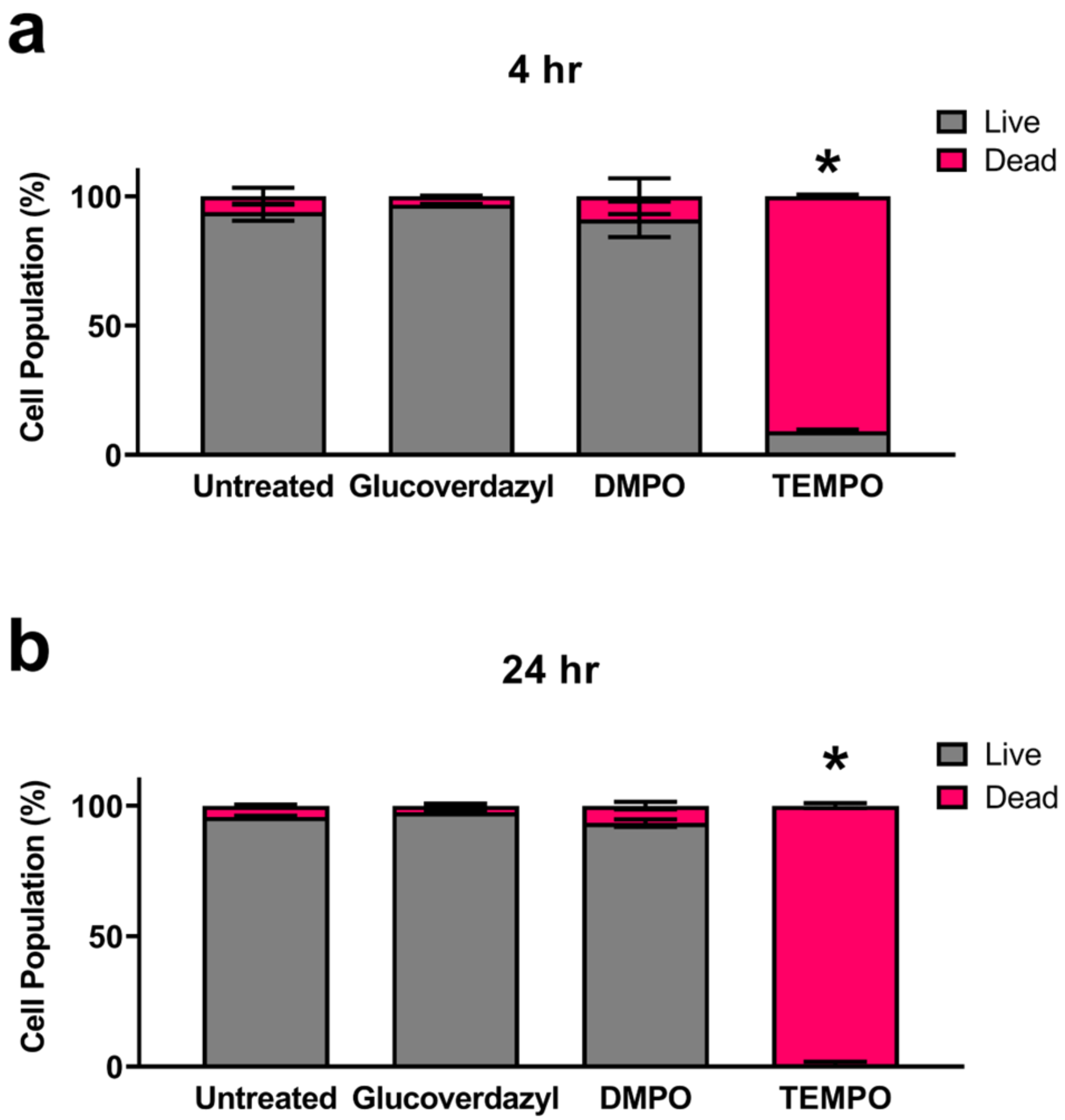

S16. Cellular viability in other radicals and spin traps. Cytotoxicity evaluation of glucoverdazyl, 5,5-dimethyl-1-pyrroline N-oxide (DMPO), and 2,2,6,6-Tetramethylpiperidin-1-yl)oxyl (TEMPO) in human renal proximal tubule cells at 10 mM concentrations after 4 hr (A) or 24 hr (B) incubations under cell culture conditions. Data are represented as means ± SD of n = 3 replicates. Statistical analysis was done by one-way ANOVA followed by a Tukey post-hoc test. * p<0.0001 of the live and dead cell populations compared to the populations of the other conditions. An example gating strategy used to obtain this data can be seen in Supplementary Figure 23.

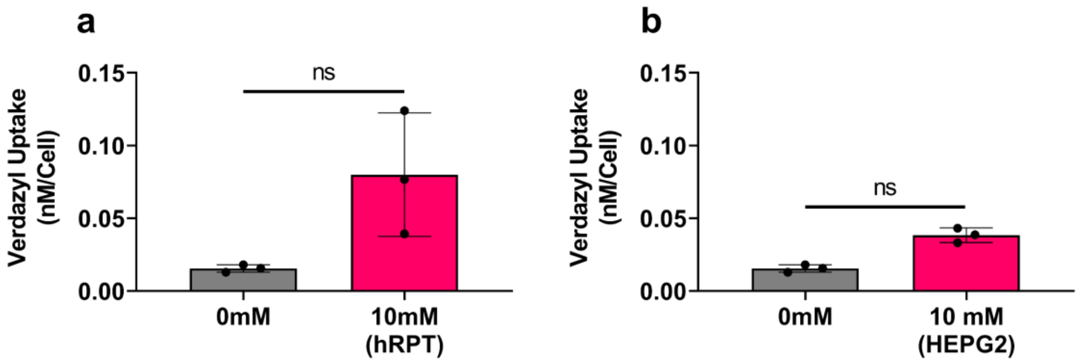

S17. Cellular internalization of glucoverdazyl. Glucoverdazyl uptake in (A) human renal proximal tubules cells (hRPT) under cell culture conditions, and (B) HepG2 hepatocarcinoma cells grown to confluency and starved for 1 hr with glucose-free media. In both experiments, uptake was measured by EPR activity after 24 hr incubation with 10 mM glucoverdazyl, as compared to a known concentration and normalized to number of cells loaded into the EPR tube. Data are represented as means ± SD of n = 3 replicates. Statistical analysis was done by one-way ANOVA followed by a Tukey post-hoc test.

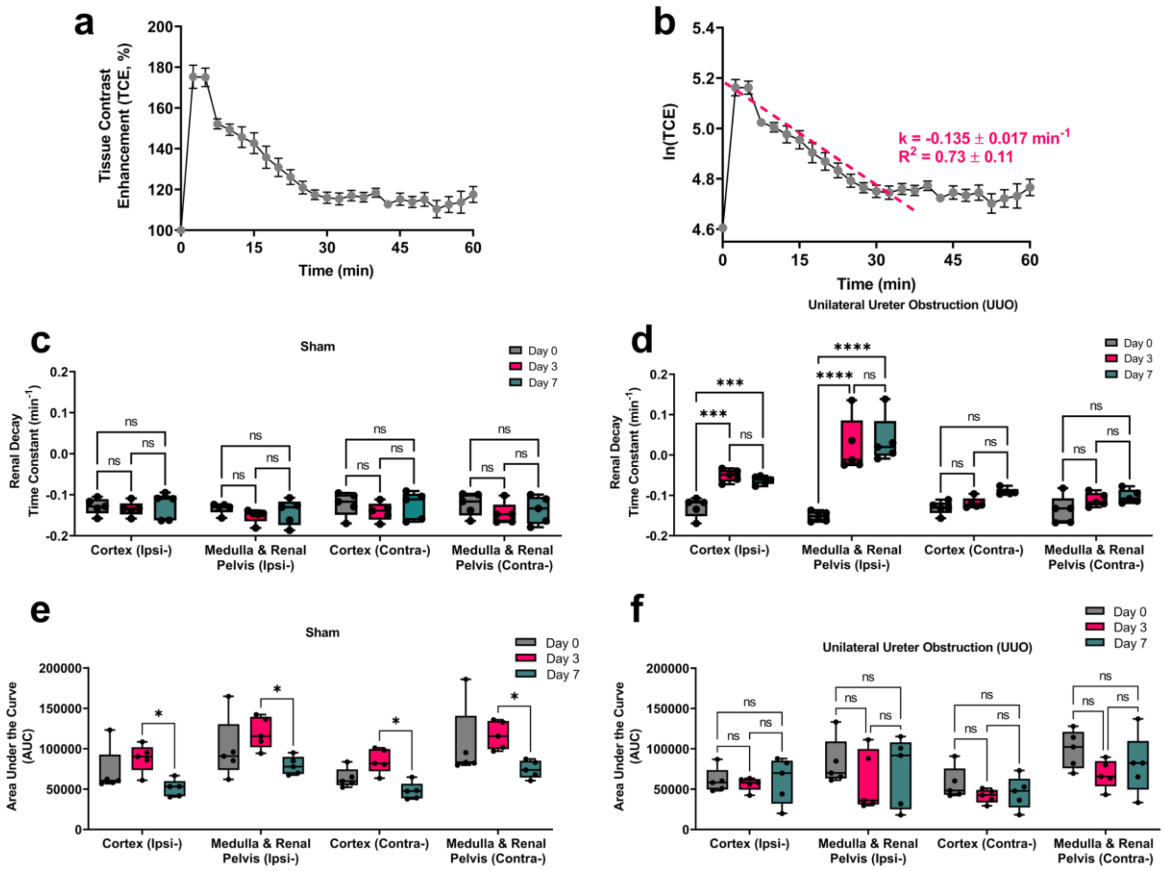

S18. Glucoverdazyl-enhanced DCE-MRI data in the unilateral ureter obstruction (UUO) model. A) Normalized intensity-over-time curves of UUO mice on day 0. Curve is presented as mean normalized intensity ± SEM of n = 10 mice (5 from sham group and 5 from UUO group). B) The semi-natural-log regression curve of A) with the line of best fit from t = 0 min to t = 40 min. Data are presented as mean RDTC (k) and R^2 value ± SD of n = 10 mice (5 from sham group and 5 from UUO group). RDTC values for the cortex and medulla & renal pelvis (MRP) regions-of-interest (ROIs) for the sham group (C) and UUO group (D). Data are presented as box-and-whisker plots of the single average RDTC value from each kidney (ipsi- or contralateral) from each region, individually, from each mouse (n = 5). *** p=0.001 (Day 0 to Day 15) and p=0.0001 (Day 0 to Day 30), **** p<0.0001. The same data and plots for AUC are shown in E) and F) for the sham and UUO groups, respectively. In E), * p=0.031 (Sham Ipsi-), * p=0.030 (UUO Ipsi-), * p=0.018 (Sham Contra-), and * p=0.013 (UUO Contra-). Statistical analysis was done by repeated measures two-way ANOVA followed by a Tukey post-hoc test. In all graphs, ns non-significant. In all boxplots, whiskers are drawn from the minimum to maximum values, box bounds represent the interquartile range, and the line within the box represents the median.

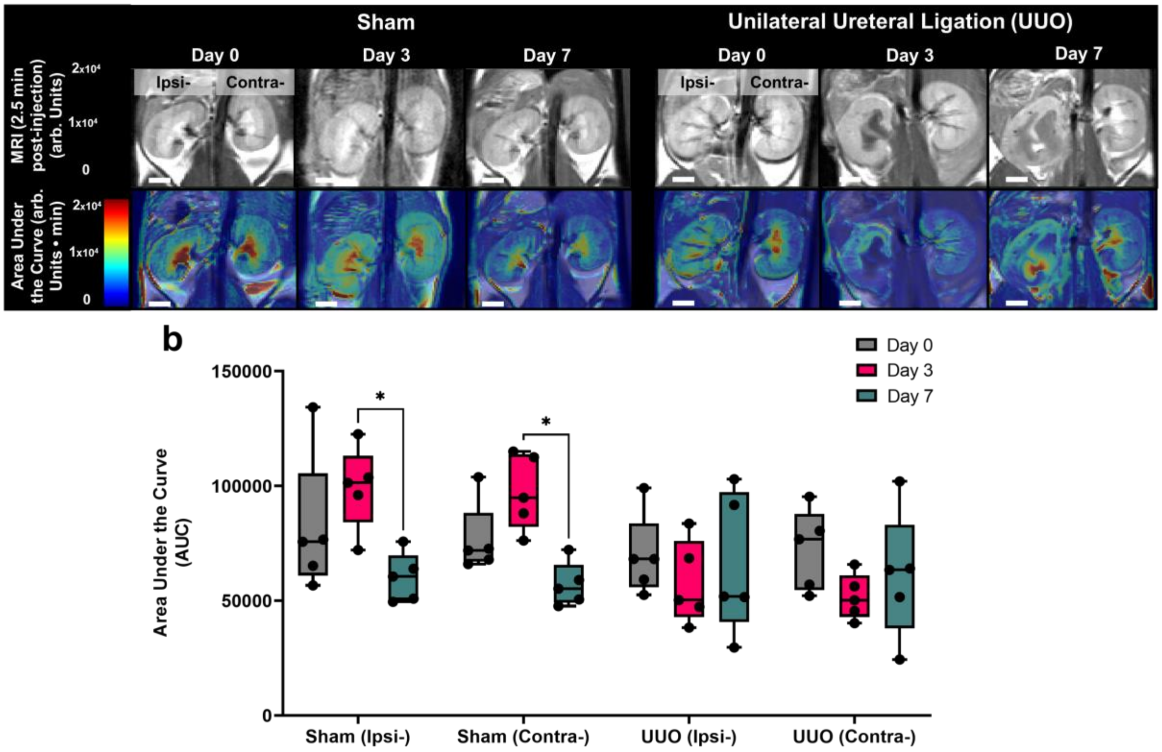

S19. Glucoverdazyl-enhanced DCE-MRI in a mouse mode of unilateral ureteral obstruction. A) T_1-weighted images of kidneys at t = 2.5 min post-injection (*top)* and AUC maps (*bottom)* for Sham and UUO groups. The white scalebar represents 2 mm. B) AUC values for each kidney at each post-injury time-point. Data are presented as box-and-whisker plots of the single average AUC value from each kidney (ipsi- or contralateral) individually, from each mouse (n = 5). * p=0.017 (Sham Ipsi-) and * p=0.013 (Sham Contra-). Statistical analysis was done by repeated measures two-way ANOVA followed by a Tukey post-hoc test. In all graphs, ns non-significant. In all boxplots, whiskers are drawn from the minimum to maximum values, box bounds represent the interquartile range, and the line within the box represents the median.

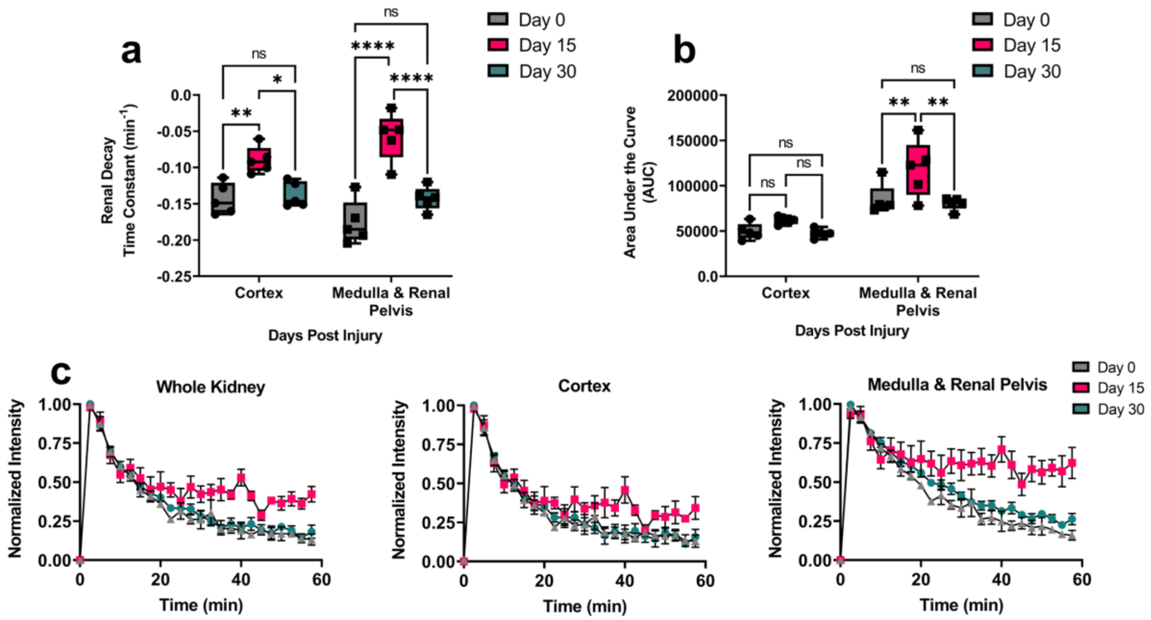

S20. Glucoverdazyl-enhanced DCE-MRI data in the folic acid nephropathy (FAN) model. A) RDTC values for the cortex and medulla & renal pelvis (MRP) regions-of-interest (ROIs). Data are presented as box-and-whisker plots of the single average RDTC value from each mouse (n = 5). * p=0.013, ** p=0.006, and **** p<0.0001. B) The same data and plots as in A) but for AUC values are shown. ** p=0.005 (Day 0 to Day 15) and ** p=0.002 (Day 15 to Day 30). C) The normalized intensity-over-time curves for the FAN model over the entire kidney, as well as for the cortex and MRP regions for each of the post injury-time points. Curves are presented as the mean normalized intensity at each time point ± SEM of n = 5 mice. Statistical analysis was done by repeated measures two-way ANOVA followed by a Tukey post-hoc test. In all graphs, ns non-significant. In all boxplots, whiskers are drawn from the minimum to maximum values, box bounds represent the interquartile range, and the line within the box represents the median.

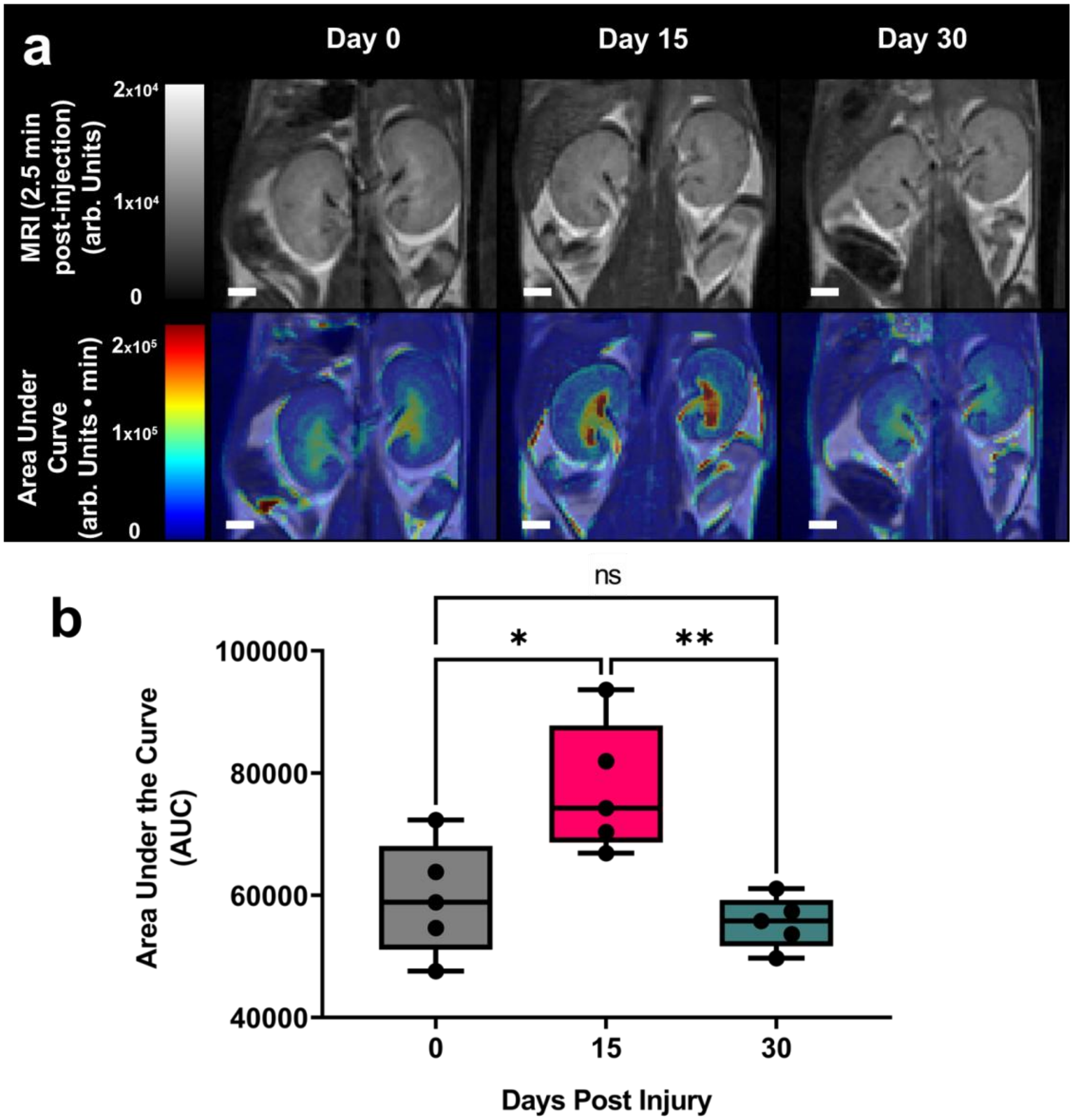

S21. Glucoverdazyl-enhanced DCE-MRI of folic acid-induced nephropathy. A) T_1-weighted images of kidneys at t = 2.5 min post-injection (*top)* and AUC maps (*bottom).* The white scalebar represents 2 mm. B) AUC values for kidneys at each post-injury time point. Data are presented as box-and-whisker plots of the single average AUC value from both kidneys of each mouse (n = 5). * p=0.014 and ** p=0.005. Statistical analysis was done by repeated measures one-way ANOVA followed by a Tukey post-hoc test. In all graphs, ns non-significant. In all boxplots, whiskers are drawn from the minimum to maximum values, box bounds represent the interquartile range, and the line within the box represents the median.

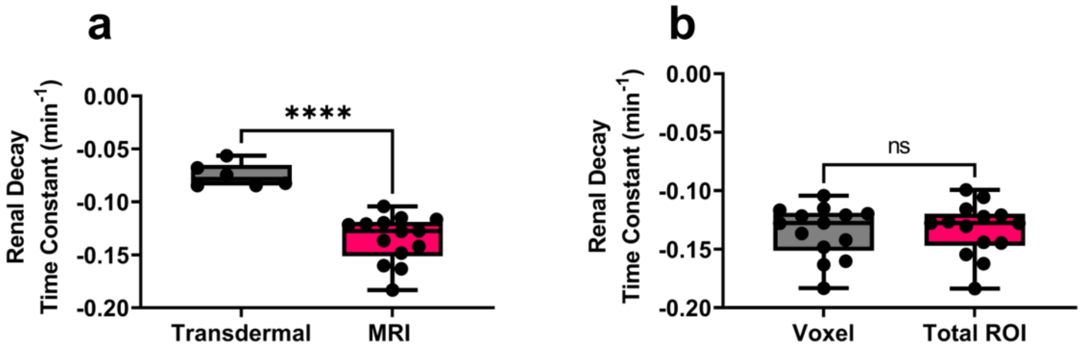

S22. Deriving the renal decay time constant in transdermal compared to MRI. A) Comparison between the transdermal fluorescence RDTC value in healthy BALB/c mice (n = 6) as determined by MediBeacon software and the RDTC value in healthy BALB/c mice (n = 14) as determined by glucoverdazyl DCE-MRI. B) A comparison between RDTC value in healthy BALB/c mice by using intensity measurements derived per voxel or from the entire ROI of a slice at each timepoint (n = 14 mice for both). Data are presented as Data are presented as box-and-whisker plots of the single RDTC (A, transdermal) or single average RDTC (A, MRI and B). Statistical analysis was done by one-way ANOVA followed by a Tukey post-hoc test. In all graphs, ns non-significant and **** $p < 0.0001$

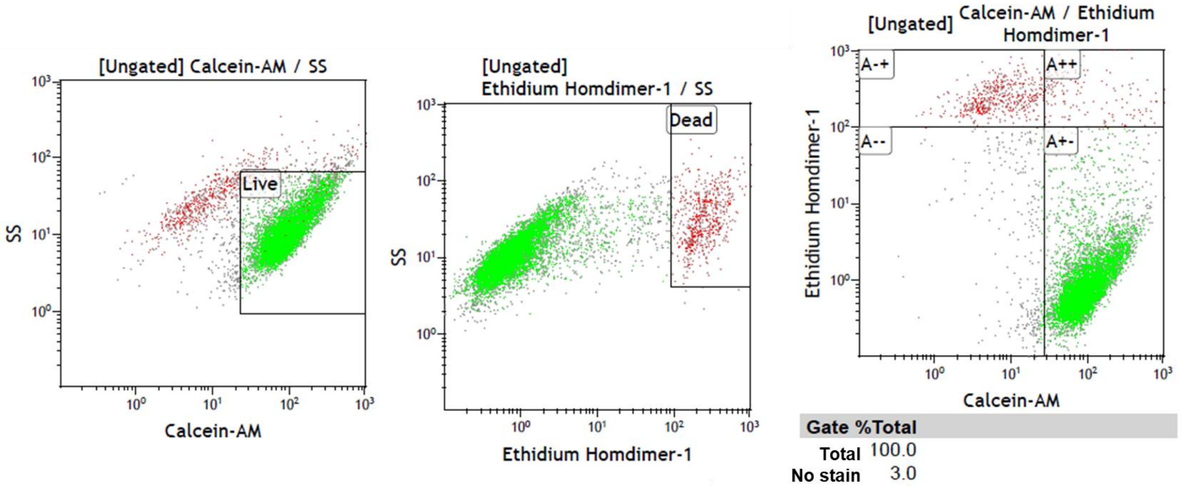

Gate	%Total
Total	100.0
No stain	3.0
Dead	4.9
Live	90.5
Double Stain	1.6

S23. Example gating strategy used for cell viability assays with flow cytometry. Example of the gating strategy used for all cell viability assays. The green region represents singly stained live cells, and the red region represents singly stained dead cells. Double or non-stained cells were not included in analysis.

4. References:

1. Castañar, L., Poggetto, G. D., Colbourne, A. A., Morris, G. A. & Nilsson, M. The GNAT: A new tool for processing NMR data. *Magnetic Resonance in Chemistry* **56**, 546–558 (2018).

2. Caravan, P. *et al.* The interaction of MS-325 with human serum albumin and its effect on proton relaxation rates. *J Am Chem Soc* **124**, 3152–3162 (2002).
