# Supplementary material for: Direct mapping of kidney function by DCE-MRI urography using a tetrazinanone organic radical contrast agent
Source: Nat Commun. 2023 Jul 5;14:3965. doi: 10.1038/s41467-023-39720-x (PMC10322853; doi:10.1038/s41467-023-39720-x)
Supplement: Supplementary file 3 — Reporting Summary [file 41467_2023_39720_MOESM3_ESM.pdf]

nature portfolio

Corresponding author(s): Adam Shuhendler

Last updated by author(s): 06/15/2023

Reporting Summary

Nature Portfolio wishes to improve the reproducibility of the work that we publish. This form provides structure for consistency and transparency in reporting. For further information on Nature Portfolio policies, see our Editorial Policies and the Editorial Policy Checklist.

Statistics

For all statistical analyses, confirm that the following items are present in the figure legend, table legend, main text, or Methods section.

n/a	Confirmed	
☐	☒	The exact sample size (*n*) for each experimental group/condition, given as a discrete number and unit of measurement
☐	☒	A statement on whether measurements were taken from distinct samples or whether the same sample was measured repeatedly
☐	☒	The statistical test(s) used AND whether they are one- or two-sided<br>*Only common tests should be described solely by name; describe more complex techniques in the Methods section.*
☒	☐	A description of all covariates tested
☐	☒	A description of any assumptions or corrections, such as tests of normality and adjustment for multiple comparisons
☐	☒	A full description of the statistical parameters including central tendency (e.g. means) or other basic estimates (e.g. regression coefficient) AND variation (e.g. standard deviation) or associated estimates of uncertainty (e.g. confidence intervals)
☐	☒	For null hypothesis testing, the test statistic (e.g. *F*, *t*, *r*) with confidence intervals, effect sizes, degrees of freedom and *P* value noted<br>*Give P values as exact values whenever suitable.*
☒	☐	For Bayesian analysis, information on the choice of priors and Markov chain Monte Carlo settings
☒	☐	For hierarchical and complex designs, identification of the appropriate level for tests and full reporting of outcomes
☒	☐	Estimates of effect sizes (e.g. Cohen's *d*, Pearson's *r*), indicating how they were calculated

Our web collection on statistics for biologists contains articles on many of the points above.

Software and code

Policy information about availability of computer code

Data collection	All data were acquired with commercial software packages, including MR Solutions Preclinical Scan, MestreNova, and TopSpin
Data analysis	Data analysis was performed using custom script for MatLab software. Statistical analyses were performed using GraphPad Prism.

For manuscripts utilizing custom algorithms or software that are central to the research but not yet described in published literature, software must be made available to editors and reviewers. We strongly encourage code deposition in a community repository (e.g. GitHub). See the Nature Portfolio guidelines for submitting code & software for further information.

Data

Policy information about availability of data

All manuscripts must include a data availability statement. This statement should provide the following information, where applicable:

- Accession codes, unique identifiers, or web links for publicly available datasets
- A description of any restrictions on data availability
- For clinical datasets or third party data, please ensure that the statement adheres to our policy

All data are available upon request. The data is raw imaging files that we would be happy to send to interested parties upon request.

Research involving human participants, their data, or biological material

Policy information about studies with human participants or human data. See also policy information about sex, gender (identity/presentation), and sexual orientation and race, ethnicity and racism.

Reporting on sex and gender	N/A
Reporting on race, ethnicity, or other socially relevant groupings	N/A
Population characteristics	N/A
Recruitment	N/A
Ethics oversight	N/A

Note that full information on the approval of the study protocol must also be provided in the manuscript.

Field-specific reporting

Please select the one below that is the best fit for your research. If you are not sure, read the appropriate sections before making your selection.

- [x] Life sciences
- [ ] Behavioural & social sciences
- [ ] Ecological, evolutionary & environmental sciences

For a reference copy of the document with all sections, see nature.com/documents/nr-reporting-summary-flat.pdf

Life sciences study design

All studies must disclose on these points even when the disclosure is negative.

Sample size	Sample size was determined according to our previous experience with preclinical imaging experiments, where animal numbers used were sufficient to detect differences in imaging signals statistically, but also adheres to the 3 R's of animal research related to reduction of animal numbers.
Data exclusions	No data were excluded.
Replication	Animal and cell-based experiments were performed more than or equal to 3 times, with the number of replicates indicated. All experimental attempts were successful. Physicochemical measurements were performed once on pure samples confirmed by NMR and mass spectrometry, and are inherent properties of the molecules under evaluation.
Randomization	Studies were longitudinal so animals served as their own healthy controls to eliminate biological variability between groups. Randomization was not necessary in this case. For experiments not involving animals, samples were taken from large synthetic batches after structure and purity confirmation by NMR and mass spectrometry, and thus randomization was not a consideration.
Blinding	Investigators were not blinded to the groups because the study was longitudinal so all animals became ill. However image analysis occurred prior to histological analyses, so histological outcomes were not known prior to imaging analysis.

Reporting for specific materials, systems and methods

We require information from authors about some types of materials, experimental systems and methods used in many studies. Here, indicate whether each material, system or method listed is relevant to your study. If you are not sure if a list item applies to your research, read the appropriate section before selecting a response.

Materials & experimental systems

n/a	Involved in the study	
x		Antibodies
	x	Eukaryotic cell lines
x		Palaeontology and archaeology
	x	Animals and other organisms
x		Clinical data
x		Dual use research of concern
x		Plants

Methods

n/a	Involved in the study	
x		ChIP-seq
	x	Flow cytometry
x		MRI-based neuroimaging

Eukaryotic cell lines

Policy information about cell lines and Sex and Gender in Research

Cell line source(s)	H460, ATCC, male<br>hRPT, ScienCell, unknown<br>HepG2, ATCC, male
Authentication	hRPT cells were authenticated by ScienCell. All other cell lines were not authenticated.
Mycoplasma contamination	All cell lines were tested negative for mycoplasma
Commonly misidentified lines (See ICLAC register)	No commonly misidentified cell lines were used in the study.

Animals and other research organisms

Policy information about studies involving animals; ARRIVE guidelines recommended for reporting animal research, and Sex and Gender in Research

Laboratory animals	8 week old female C57Bl/6 (UUO) and 8 week old female Balb/C mice (FAN). The following was added to the SI: Mice were housed under 12 hr light/dark cycle, and ambient temperature of 20-24°C and 45 to 65% humidity.
Wild animals	No wild animals were used in the study.
Reporting on sex	Sex was not considered in the current study design. Sex was rather chosen based on survivability of induced animal models.
Field-collected samples	No field collected samples were used in this study.
Ethics oversight	All animal studies were conducted under Animal Use Protocol HIe-3640-R1 approved by the IACUC at the University of Ottawa.

Note that full information on the approval of the study protocol must also be provided in the manuscript.

Flow Cytometry

Plots

Confirm that:

- [x] The axis labels state the marker and fluorochrome used (e.g. CD4-FITC).
- [x] The axis scales are clearly visible. Include numbers along axes only for bottom left plot of group (a 'group' is an analysis of identical markers).
- [x] All plots are contour plots with outliers or pseudocolor plots.
- [x] A numerical value for number of cells or percentage (with statistics) is provided.

Methodology

Sample preparation	Cellswere grown in RPMI-1640 (RPMI) media supplemented with 10% fetal bovine serum (FBS) and 1% penicillin-streptomycin (P/S) until 80% confluent, at which point they were passaged. Cells were passaged three times before being seeded in to a 6-well plate and grown until 80% confluent. Cells were seeded to have triplicate wells of each condition. Cells were then incubated in their regular media supplemented with 0 mM, 2.5 mM, 5 mM, or 10 mM glucoverdazyl, for either 4 hrs or 24 hrs. At the respective time points, the media was aspirated and cells were washed three times with 37oC Dulbecco's phosphate-buffered saline (PBS). Afterwards, cells were lifted with trypsin-EDTA, centrifuged at 400 xg (5 min, 4oC), aspirated, then resuspended in a 1 mL PBS solution containing 0.2 µM calcein-acetoxymethyl ester (fluorescently staining live cells green) and 16 µM ethidium homodimer-1 (fluorescently staining dead cells red).
Instrument	Beckman-Coulter Gallios Flow Cytometer
Software	Kaluza
Cell population abundance	No sorting was performed
Gating strategy	Live/Dead assay is a commercial kit and gating was performed as described in the instructions. Untreated cells were stained with both Calcein-AM and ethidium homodimer-1, and the boundary of FITC-channel and TRITC-channel were defined by this sample.

- [x] Tick this box to confirm that a figure exemplifying the gating strategy is provided in the Supplementary Information.
